# The LEG program promotes the development of physical activity and fundamental movement skills in preschool children aged 3–6 years: a Delphi study

**DOI:** 10.3389/fpubh.2025.1521878

**Published:** 2025-03-25

**Authors:** Zuozheng Shi, Xi Long, Xulin Yang, Jingang Fan, Jun Tang

**Affiliations:** ^1^Early Childhood Sports and Health Research Centre, Chongqing Preschool Education College, Chongqing, China; ^2^Department of Physical Education, Sichuan International Studies University, Chongqing, China; ^3^Chongqing City Fuling District Kindergarten, No. 19, Chongqing, China

**Keywords:** LEG program, fundamental movement skills, physical activity, preschoolers, early childhood physical education programs

## Abstract

**Background:**

Effectively addressing physical inactivity and the delayed development of fundamental movement skills in preschool children aged 3–6 years.

**Methods:**

We assembled an interdisciplinary team of experts to systematically validate the LEG program indicators using the Delphi method. This Delphi study thoroughly and meticulously explored the insights of experts in the field to identify the necessary indicators of the LEG program’s role in promoting the sustainable development of physical activity and fundamental movement skills in preschoolers aged 3–6. Using a 5-point Likert scale and Wilcoxon statistical techniques, this study examines the dynamic consensus among experts and elucidates potential differences in their views.

**Results:**

After three rounds of Delphi surveys, O1, O2, and O3 reached consensus in all three rounds. T5, I5, I14, C11, and C12 disagreed due to disciplinary differences, while C18 and C22 disagreed due to cultural differences. Finally, the LEG program indicators achieved consensus on three objectives, seven tasks, 17 indicators, and 25 content areas. The results of this study strongly convey the experts’ positive perceptions of the LEG program indicators in promoting sustainable development of physical activity and fundamental movement skills in preschoolers aged 3–6 years.

**Conclusion:**

This indicates that the LEG-structured curriculum indicators we developed are scientific and reliable, aligning with the physical and mental development of preschoolers aged 3–6 years. This understanding fosters the in-depth integration of early childhood physical education and preschool education, providing a foundation for enhancing the sustainable development of physical activity and fundamental movement skills among preschoolers aged 3–6 years.

## 1 Introduction

Preschool institutions play a crucial role in promoting physical activity among children under 5 years of age. However, these children spend most of their time sedentary (50 to 94%), with only a small portion of their time engaged in low physical activity (5 to 27%) or moderate physical activity (1 to 17%) (1). This level of activity is far below the World Health Organization’s recommendation that children aged 1–4 years require at least 180 min of physical activity of varying intensities per day, while children aged 3–4 years require at least 60 min of moderate to vigorous intensity physical activity (2). Children’s health has become a significant concern in global public health, indicating that there is no time to lose in improving their physical activity levels. POITRAS VJ et al. concluded that physical activity in early childhood is closely related to body composition, cardiorespiratory endurance, bone development, fundamental movement skills, and psychological well-being (3). Moreover, physical inactivity in children may increase the risk of heart disease, hypertension, and obesity-related illnesses in adulthood (4), which suggests that physical activity has a direct impact on children’s health. Clark et al. believe that preschool children between the ages of 3 and 6 years are in a critical period for developing fundamental movement skills (5, 6) and that mastering these skills during this stage will enable them to adapt flexibly to different sports and environments throughout their lives, enhancing their willingness to participate in physical activity independently.

JONES D et al. demonstrated the correlation between fundamental movement skills and physical activity (7). Robinson et al. argue that children’s fundamental movement skills do not develop naturally but need to be taught, practiced, and reinforced through rational movement patterns (8–10), which shows that children’s mastery of fundamental movement skills must progress through at least the stages of learning movement, practicing movement, and engaging in game activities, requiring a certain level of physical activity as a prerequisite. Wick et al. concluded that participation in systematic, organized, and targeted physical activities is more effective for improving children’s fundamental movement skills than free play (11, 12), highlighting the role of a structured curriculum in enhancing fundamental movement skills and physical activity among preschoolers aged 3–6 years.

Although structured programs such as SKIP, CHAMP, SPARK, and ESPEC have been promoted globally, they do not appear to have successfully addressed the issue of inadequate physical activity levels and the delayed development of fundamental movement skills in preschoolers aged 3–6 years. We believe that a structured curriculum should be developed to foster the simultaneous growth of physical activity and fundamental movement skills in preschool children of the same age. Drawing from physical literacy theory, motor development theory, cognitive development theory, and game staging theory, we aimed to design a LEG program (L = Learning Movement; E = Exercising Movement; G = Game Activity). This structured curriculum is specifically tailored for preschool children aged 3–6 years and is based on optimizing the curriculum model of teaching, exercising, and competing in youth sports to achieve the goal of “enjoying fun, strengthening physical fitness, improving personality, and enhancing the quality of mind.” It consists of five segments: preparatory activities, learning movement(L), exercise movement(E), game activities(G), and relaxation activities, all of which promote increased levels of physical activity and the development of fundamental movement skills in children. Studies have indicated that this model effectively supports quality development in children and youth sports (13) and has demonstrated success in physical form, motor skills, and endurance (14). We found that the optimized model (LEG curriculum prototype) had high participation rates among 3- to 4-year-olds in two public classes focused on physical activity for young children in villages. This leads us to believe that the LEG curriculum model is applicable to preschoolers and highlights the necessity to validate and disseminate the curriculum indicator system.

Therefore, we attempted to develop a curriculum indicator system for the LEG program from an interdisciplinary perspective that combines kinesiology and preschool education. We employed the Delphi method for systematic demonstration to enhance the sustainable development of physical activity and fundamental movement skill levels in preschoolers aged 3–6 years.

## 2 Materials and methods

**Figure 1:**
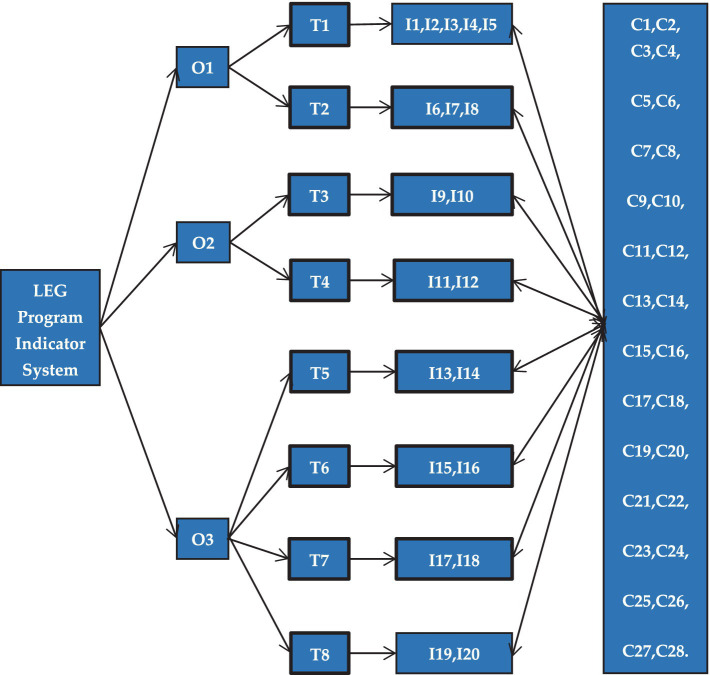
Research framework for LEG program indicator system.

In this study, the revisionist Delphi technique was utilized to organize the collection and presentation of relevant information about the area of specialization, with the goal of achieving consensus among experts in the field (15, 16). This qualitative method aims to enable a group of experts to reach an agreement on a specific topic (17, 18). First, the LEG curriculum indicator system was examined separately through group discussions to identify issues, resulting in a strong consensus. Second, an expert panel was formed, and the research process was detailed. Finally, experts were invited to explore the potential contributions of the LEG Curriculum Indicator System to the holistic and sustainable development of preschoolers aged 3–6 years and to seek consensus on the LEG Curriculum Indicator System. Figure 1 illustrates the research framework.

**Figure 2:**
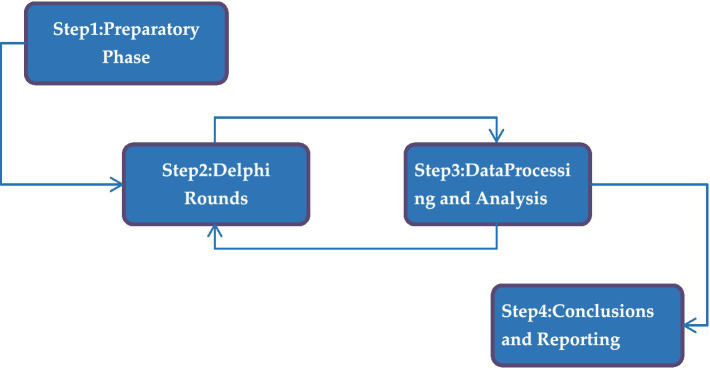
The Delphi research path.

The LEG program indicator system depicted in Figure 1 is the result of an optimization process based on the existing research framework of structured courses, including SKIP, CHAMP, SPARK, and ESPEC. The letter “O” stands for objective, “T” for task, “I” for indicator, and “C” for content. For example, “T2” associated with the curriculum objective “O1” denotes a specific goal, with I6 related to body mobility skills, I7 to object control skills, I8 to body stability skills, and C1 through C28 covering activities such as running, jumping, racket skills, and kicking a ball, among others, all aimed at achieving objectives at various levels. The current study employed the Delphi method, which involved three rounds of data collection and application. The first phase included preparation, during which new definitions and concepts were validated and reached a consensus. The subsequent phase involved implementation, featuring three visits from experts. The third phase covered data processing and analysis, wherein the collected data were examined through both quantitative and qualitative methods. Finally, the final phase consisted of reporting results and conclusions, which included discussing the findings and deriving conclusions from the study. Figure 2 shows the research process.

### 2.1 Preparation phase

This research tool incorporates concepts and content from structured courses such as SPARK, CHAMP, SKIP, and ESPEC to ensure that the subject matter is understood within current scholarship and applied practice. The Delphi method typically requires between 15 and 50 experts (19), and the selection of experts is a crucial measure of the validity of a Delphi study (20), the results of which rely heavily on the subjective insights and perspectives of the panelists (21). The Delphi research approach addresses the healthy and sustainable development of preschoolers aged 3–6 years through an interdisciplinary application of kinesiology and preschool education. The establishment of the research expert panel was carefully designed, with selection criteria favoring scholars and educators with relevant disciplinary backgrounds in kinesiology, preschool education, and research on the future development trends of early childhood physical education programs. Following an in-depth assessment of the candidates’ academic backgrounds and practical experiences, the panel of experts was identified as those with insights into applied research on physical education curricula, fundamental motor skills, and physical activity for preschoolers aged 3–6 years, and the ability to make a substantial contribution to this study. The panel not only possessed in-depth insights into the healthy and sustainable development of 3- to 6-year-old preschoolers but also maintained a multidimensional perspective that ensured the scientific validity of this study through rigorous selection criteria. This rigorous selection process brought together researchers from various regions, organizations, and positions, and the expert profiles are shown in Table 1.

**Table 1:** Summary of experts’ characteristics.

Characteristics	Round 1 (*n* = 22), *n* (95.65%)	Round 2 (*n* = 21), *n* (95.45%)	Round 3 (*n* = 20), *n* (95.24%)
Gender			
Male	14 (63.64%)	14 (66.67%)	14 (70.00%)
Female	8 (36.36%)	7 (33.33%)	6 (30.00%)
Total	22	21	20
Academic qualifications			
Bachelor’s degree	9 (40.91%)	8 (38.1%)	8 (40.00%)
Master’s degree	8 (36.36.91%)	8 (38.1%)	8 (40.00%)
Ph.D. degree	5 (22.72%)	5 (23.80%)	4 (20.00%)
Total	22	21	20
Field of research			
Kinesiology	9 (40.91%)	10 (47.62%)	10 (50.00%)
Pre-primary education	13 (59.09%)	11 (52.38%)	10 (50.00%)
Total	22	21	20
Years of experience			
1-5 years	4 (18.18%)	5 (23.81%)	5 (25.00%)
6-10 years	6 (27.27%)	6 (28.57%)	5 (25.00%)
11-15 years	7 (31.82%)	5 (23.81%)	6 (30.00%)
Over 15 years	5 (22.73%)	5 (23.81%)	4 (20.00%)
Total	22	21	20
Working organization			
Research organization(College)	15 (68.18%)	14 (66.67%)	14 (70.00%)
Early education organization	5 (22.73%)	5 (23.81%)	4 (20.00%)
Educational and training institutions	2 (9.09%)	2 (9.52%)	2 (10.00%)
Total	22	21	20

### 2.2 Delphi rounds

A Delphi methodology was employed to explore the LEG curriculum indicator system. To achieve this goal, we conducted a structured, iterative group consultation process consisting of three rounds of surveys to determine consensus among experts. In each survey round, we analyzed the experts’ views, listened to their suggestions and feedback, optimized the survey instrument by adding, revising, removing, and incorporating changes, and continuously improved the survey items to enhance scientific rigor and reliability as a research tool. Our approach involved carrying over all questions from each questionnaire round to the next, including previously reached consensus opinions. We defined “agreement” as when more than 75% of researchers rated an opinion with a score of 4 or 5, indicating agreement (16). In each round, the panel of experts had the opportunity to revise their responses, ensuring the stability of their answers and revealing consensus and disagreement regarding the sustainable development of preschoolers’ health, aged 3–6 years until consensus on the research topic was achieved. Due to the interdisciplinary nature of the LEG curriculum indicator system for the healthy and sustainable development of preschoolers, we primarily used closed-ended questions supplemented by open-ended questions. The former generated objective data for quantitative analysis, while the latter allowed for the expression of innovative ideas, enriching the dimensions of the research tool and laying the foundation for its revision.

### 2.3 Data processing and analysis

The data were analyzed using Excel 2024 and SPSS 29.0 software for descriptive statistics. A 5-point Likert scale was employed to evaluate the data by assigning scores from 1 to 5, ranging from “unimportant” to “very important.” The median and interquartile range (IQR) of each 5-point Likert question response were calculated. We followed the recommendations of Heiko (22) to reach a consensus. In this context, agreement with an item was considered to have been reached when the IQR of the participants’ responses to that item in the round was ≤1. The IQR is typically regarded as a suitable criterion for consensus in 4- or 5-point scales. Based on this criterion, we defined “agreement” with an item in a given round as occurring when the IQR of the participants’ responses was ≤1 and defined “disagreement” otherwise. A statistically significant difference between the rounds was tested. We used the Wilcoxon matched-pairs signed rank test to assess the stability of these responses, which is commonly utilized to evaluate response stability in two consecutive rounds in Delphi studies. According to these criteria, we considered that participants’ responses to an item in two consecutive rounds were stable when the results of the Wilcoxon matched-pairs signed rank test did not indicate a statistically significant difference and deemed them unstable otherwise (16). The survey was concluded when agreement was reached on all items, no new items were identified, and the non-agreed items demonstrated stability over two consecutive rounds.

### 2.4 Concluding and reporting

Our study is reported in the conclusion and discussion, and the results of this study include all statements and information regarding judgment, consensus, and stability.

## 3 Results

In the first round of the survey, a total of 23 invitations were sent out, of which 22 participants took part (95.65%). In the second round of the survey, a total of 22 invitations were sent out, of which 21 participants took part (95.45%). In the third round of the survey, a total of 21 invitations were sent out, of which 20 participants took part (95.24%).

### 3.1 Round one

The first round of the Delphi study collected responses from a panel of experts on the LEG curriculum indicator system, with the results presented in Table 2. The validity and reliability of the research instrument were assessed using Cronbach’s alpha (*α* = 0.970), which indicated high validity and reliability. In this round, a consensus was reached on 53 indicators. However, the panel objected (<75% agreement) to the following indicators: sense of competition (T5), quality of endurance (I5), daring to take risks (I14), muscular endurance (C12), slide (C18), and throw the ball (C22). The panel also identified redundancies, arguing that I13 (courage to challenge) overlapped with I14 (dare to take risks), I15 (respect for order) with I16 (respect for discipline), I19 (teamwork spirit) with I20 (willingness to cooperate), C15 (skip) with C27(Leap), and C22 (throw the ball) with C23 (throw a ball). Additionally, I18(sense of responsibility) was considered extraneous, and the T4 indicator was noted as missing. Based on these concerns, some modifications were suggested.

**Table 2:** Results of round one of the Delphi study.

Items	Agreement or disagreement	Scores of 4 or 5 (*n* = 22), *n* (%)	Scores of 5 (*n* = 22), *n* (%)	Results
Objectives (O)				
O1.Physical capability	Agreement	21 (95.5)	15 (68.2)	Reservation
O2.Healthy behaviors	Agreement	21 (95.5)	12 (54.5)	Reservation
O3.Motor cognition	Agreement	19 (86.4)	8 (36.4)	Reservation
Tasks (T)				
T1.Physical fitness	Agreement	19 (86.4)	15 (68.2)	Reservation
T2.Motor skills	Agreement	18 (81.8)	11 (50)	Reservation
T3.Body health	Agreement	20 (90.9)	16 (72.7)	Reservation
T4.Psychological health	Agreement	19 (86.4)	15 (68.2)	Reservation
T5.Competitive awareness	Disagreement	15 (68.2)	7 (31.8)	Remove
T6.Rule awareness	Agreement	20 (90.9)	14 (63.6)	Reservation
T7.Safety awareness	Agreement	21 (95.5)	17 (77.3)	Reservation
T8.Teamwork awareness	Agreement	21 (95.5)	15 (68.2)	Reservation
Indicators (I)				
I1.Body coordination	Agreement	20 (90.9)	17 (77.3)	Reservation
I2.Quality of velocity	Agreement	17 (77.3)	10 (45.5)	Reservation
I3.Balance	Agreement	20 (90.9)	15 (68.2)	Reservation
I4.Quality of strength	Agreement	17 (77.3)	9 (40.9)	Reservation
I5.Quality of endurance	Disagreement	15 (68.2)	9 (40.9)	Reservation
I6.Body movement skills	Agreement	21 (95.5)	16 (72.7)	Reservation
I7.Object control skills	Agreement	21 (95.5)	14 (63.6)	Reservation
I8.Body stability skills	Agreement	19 (86.4)	14 (63.6)	Reservation
I9.Physical activity	Agreement	20 (90.9)	12 (54.5)	Reservation
I10.Motor behavior	Agreement	19 (86.4)	12 (54.5)	Reservation
I11.Emotional mastery	Agreement	21 (95.5)	14 (63.6)	Revise
I12.Pro-social behavior	Disagreement	19 (86.4)	12 (54.5)	Remove
I13.Courage to challenge	Agreement	20 (90.9)	14 (63.6)	Revise
I14.Dare to take risks	Disagreement	15 (68.2)	10 (45.5)	Remove
I15.Respect for order	Agreement	22 (100)	18 (81.8)	Incorporation
I16.Respect for discipline	Agreement	20 (90.9)	14 (63.6)	Incorporation
I17.Self-protection	Agreement	22 (100)	19 (86.4)	Reservation
I18.Sense of responsibility	Disagreement	18 (81.8)	10 (45.5)	Remove
I19.Teamwork spirit	Agreement	21 (95.5)	12 (54.5)	Incorporation
I20.Willingness to cooperate	Agreement	22 (100)	15 (68.2)	Incorporation
Contents (C)				
C1.Hand-eye coordination	Agreement	21 (95.5)	15 (68.2)	Reservation
C2.Hand-foot coordination	Agreement	20 (90.9)	14 (63.6)	Reservation
C3.Reaction velocity	Agreement	19 (86.4)	12 (54.5)	Reservation
C4.Displacement velocity	Agreement	19 (86.4)	8 (36.4)	Reservation
C5.Velocity of body movement	Agreement	20 (90.9)	10 (45.5)	Reservation
C6.Dynamic balance	Agreement	21 (95.5)	15 (68.2)	Reservation
C7.Static balance	Agreement	19 (86.4)	12 (54.5)	Reservation
C8.Upper body Strength	Agreement	17 (77.3)	9 (40.9)	Reservation
C9.Lumbar and abdominal strength	Agreement	20 (90.9)	12 (54.5)	Reservation
C10.Lower body strength	Agreement	19 (86.4)	11 (50)	Reservation
C11.Cardiorespiratory endurance	Agreement	17 (77.3)	10 (45.5)	Reservation
C12.Muscle endurance	Disagreement	15 (68.2)	8 (36.4)	Remove
C13.Walk	Agreement	21 (95.5)	11 (50)	Reservation
C14.Run	Agreement	21 (95.5)	15 (68.2)	Reservation
C15.Skip	Agreement	21 (95.5)	14 (63.6)	Incorporation
C16.Climb	Agreement	20 (90.9)	13 (59.1)	Reservation
C17.Straddle	Agreement	21 (95.5)	9 (40.9)	Reservation
C18.Slide	Disagreement	16 (72.7)	7 (31.8)	Reservation
C19.Racket the ball	Agreement	21 (95.5)	9 (40.9)	Reservation
C20.Hit the ball	Agreement	17 (77.3)	8 (36.4)	Reservation
C21.Passing and receiving the ball	Agreement	17 (77.3)	5 (22.7)	Reservation
C22.Throw the ball	Disagreement	16 (72.7)	8 (36.4)	Incorporation
C23.Throwing a ball	Agreement	17 (77.3)	6 (27.3)	Incorporation
C24.Kick the ball	Agreement	18 (81.8)	8 (36.4)	Reservation
C25.Roll	Agreement	17 (77.3)	10 (45.5)	Reservation
C26.Whirl	Agreement	17 (77.3)	10 (45.5)	Reservation
C27.Leap	Agreement	20 (90.9)	12 (54.5)	Incorporation
C28.Hedge	Agreement	22 (100)	13 (59.1)	Reservation

Regarding the quality of endurance (I5) indicator, it was retained because the World Health Organization (WHO) recommends that children aged 1–4 years engage in at least 180 min of physical activity of varying intensities per day, with children aged 3–4 years requiring at least 60 min of moderate-to-vigorous-intensity physical activity (2). Achieving these activity levels requires a certain degree of cardiorespiratory endurance, justifying its inclusion.

As for the slide (C18) and throw the ball (C22) indicators, initial objections were due to a lack of awareness among preschool pedagogy experts regarding the significance of these fundamental movement skills in competitive sports programs. After discussions with the experts, where their importance was explained along with supporting research cases, these indicators were retained for the second round of the Delphi survey.

Based on experts’ suggestions, in the second round of the survey, we incorporated I13 and I14 into daring to challenge (I13), I15 and I16 for respect for order (I15), I19 and I20 into willingness to cooperate (I17), C15 and C27 into skip (C14), and C22 and C23 into throw the ball (C21). Additionally, we removed three irrelevant indicators (T5, C12, and I18) and added emotional mastery (I11), self-recognition (I12), friendly competition (I13), movement instructions (C26), and musical rhythm (C27).

### 3.2 Round two

This round of research included the experts and topics from the first round of the Delphi study. Cronbach’s alpha coefficient (*α* = 0.977) indicated the high reliability of the second-round research instruments. The results of this round are shown in Table 3. We added the following indicators: emotional mastery (I11,90.5%), self-recognition (I12,85.7%), friendly competition (I14,90.5%), movement instruction (C26,80.9%), musical rhythms (C27,100%). Additionally, I13 was revised to dare to challenge (85.7%), all of which achieved a strong consensus (≧75%).

**Table 3:** Results of round two of the Delphi study.

Items	Agreement or disagreement	Scores of 4 or 5 (*n* = 21), *n* (%) (*n*)	Scores of 5 (*n* = 21), *n* (%)	Stability
Objectives (O)				
O1.Physical capability	Agreement	19 (90.5)	16 (76.2)	YES
O2.Healthy behaviors	Agreement	21 (100)	15 (71.4)	YES
O3.Motor cognition	Agreement	18 (85.7)	11 (52.4)	YES
Tasks (T)				
T1.Physical fitness	Agreement	20 (95.2)	16 (81)	YES
T2.Motor skills	Agreement	17 (80.9)	10 (47.6)	YES
T3.Body health	Agreement	20 (95.2)	15 (71.4)	YES
T4.Psychological health	Agreement	20 (95.2)	16 (81)	YES
T5.Rule awareness	Agreement	18 (85.7)	14 (66.7)	YES
T6.Safety awareness	Agreement	20 (95.2)	19 (90.5)	YES
T7.Teamwork awareness	Agreement	20 (95.2)	9 (42.9)	YES
Indicators (I)				
I1.Body coordination	Agreement	20 (95.2)	14 (66.7)	YES
I2.Quality of velocity	Disagreement	16 (76.2)	8 (38.1)	YES
I3.Balance	Agreement	18 (85.7)	14 (66.7)	YES
I4.Quality of strength	Disagreement	16 (76.2)	7 (33.3)	YES
I5.Quality of endurance	Agreement	14 (66.6)	4 (19)	YES
I6.Body movement skills	Agreement	20 (95.2)	12 (57.1)	YES
I7.Object control skills	Agreement	19 (90.5)	11 (52.4)	YES
I8.Body stability skills	Agreement	18 (85.7)	11 (52.4)	YES
I9.Physical activity	Agreement	21 (100)	14 (66.7)	YES
I10.Motor behavior	Agreement	18 (85.7)	11 (52.4)	YES
I11.Emotional mastery	Agreement	19 (90.5)	13 (61.9)	N/A
I12.self-recognition	Agreement	18 (85.7)	10 (47.6)	N/A
I13.Dare to challenge	Agreement	18 (85.7)	14 (66.7)	N/A
I14.Friendly competition	Agreement	19 (90.5)	9 (42.9)	N/A
I15.Respect for order	Agreement	18 (85.7)	16 (76.2)	YES
I16.Self-protection	Agreement	20 (95.2)	18 (85.7)	YES
I17.Willingness to cooperate	Agreement	17 (80.9)	7 (33.3)	NO (0.008)
Contents (C)				YES
C1.Hand-eye coordination	Agreement	21 (100)	16 (76.2)	YES
C2.Hand-foot coordination	Agreement	21 (100)	12 (57.1)	YES
C3.Reaction velocity	Agreement	19 (90.5)	7 (33.3)	YES
C4.Displacement velocity	Disagreement	16 (76.2)	9 (42.9)	YES
C5.Velocity of body movement	Disagreement	15 (71.4)	9 (42.9)	NO (0.032)
C6.Dynamic balance	Agreement	19 (90.5)	13 (61.9)	YES
C7.Static balance	Agreement	17 (80.9)	10 (47.6)	YES
C8.Upper body Strength	Disagreement	16 (76.2)	8 (38.1)	YES
C9.Lumbar and abdominal strength	Disagreement	14 (66.7)	5 (23.8)	YES
C10.Lower body strength	Agreement	18 (85.7)	7 (33.3)	YES
C11.Cardiorespiratory endurance	Agreement	18 (85.7)	8 (38.1)	YES
C12.Walk	Agreement	20 (95.2)	17 (80.9)	YES
C13.Run	Agreement	21 (100)	18 (85.7)	NO (0.02)
C14.Skip	Agreement	21 (100)	18 (85.7)	NO (0.005)
C15.Climb	Agreement	19 (90.5)	14 (66.7)	YES
C16.Straddle	Agreement	18 (85.7)	11 (52.4)	YES
C17.Slide	Agreement	16 (76.2)	5 (23.8)	NO (0.039)
C18.Racket the ball	Agreement	18 (85.7)	12 (57.1)	YES
C19.Hit the ball	Disagreement	16 (76.2)	8 (38.1)	YES
C20.Passing and receiving the ball	Agreement	17 (80.9)	5 (23.8)	YES
C21.Throwing the ball	Agreement	17 (80.9)	10 (47.6)	YES
C22.Kick the ball	Agreement	18 (85.7)	9 (42.9)	YES
C23.Roll	Agreement	20 (95.2)	9 (42.9)	YES
C24.Whirl	Agreement	18 (85.7)	7 (33.3)	YES
C25.Hedge	Agreement	18 (85.7)	8 (38.1)	YES
C26.Movement instruction	Agreement	17 (80.9)	9 (42.9)	N/A
C27.Music rhythm	Agreement	21 (100)	13 (61.9)	N/A

However, consistency tests for I2, I4, C4, C5, C8, C9, and C19 were found to be non-compliant (IQR≧1) in two consecutive rounds. Based on existing research, the indicators velocity of body movement(C5)and waist and abdominal strength(C9)were removed, while the following indicators were retained: I2(quality of velocity), I4(quality of strength), C4(displacement velocity), C8(upper body strength), and C19(hit the ball). The results of the second-round survey showed that the LEG course indicator system achieved good consistency and stability across two rounds of the Delphi study, resulting in a total of 47 indicators. However, five indicators still require further investigation and validation in the third round.

### 3.3 Round three

This round of research included experts and topics from the second round of Delphi research. The Cronbach’s alpha coefficient (*α* = 0.988) indicated the high reliability of the instrument in the third round. The results of the third round of the Delphi study are shown in Table 4. In the third round of the survey, the consistency test for I2, I4, C4, C8, and C19 (≧75%) demonstrated good stability, indicating that the expert panel reached a consensus. The LEG curriculum indicator system achieved good consistency and stability across all three rounds of the Delphi survey, resulting in three objectives, seven tasks, 17 indicators, and 25 content items, with no additional items proposed by the panel. Consequently, the survey was concluded.

**Table 4:** Results of round three of the Delphi study.

Items	Agreement or disagreement	Scores of 4 or 5(n = 20), n (%) (n)	Scores of 5 (n = 20), n (%)	Stability
Objectives (O)				
O1.Physical capability	Agreement	18 (90)	15 (75)	YES
O2.Healthy behaviors	Agreement	19 (95.5)	15 (75)	YES
O3.Motor cognition	Agreement	19 (95.5)	11 (55)	YES
Tasks (T)				
T1.Physical fitness	Agreement	18 (90)	17 (85)	YES
T2.Motor skills	Agreement	17 (85)	10 (50)	YES
T3.Body health	Agreement	19 (95.5)	15 (75)	YES
T4.Psychological health	Agreement	18 (90)	15 (75)	YES
T5.Rule awareness	Agreement	18 (90)	14 (70)	YES
T6.Safety awareness	Agreement	19 (95.5)	18 (90)	YES
T7.Teamwork awareness	Agreement	19 (95.5)	9 (45)	YES
Indicators (I)				
I1.Body coordination	Agreement	19 (95.5)	14 (70)	YES
I2.Quality of velocity	Agreement	16 (80)	8 (40)	YES
I3.Balance	Agreement	19 (95.5)	14 (70)	YES
I4.Quality of strength	Disagreement	15 (75)	8 (40)	YES
I5.Quality of endurance	Agreement	15 (75)	3 (15)	YES
I6.Body movement skills	Agreement	18 (90)	12 (60)	YES
I7.Object control skills	Agreement	18 (90)	11 (55)	YES
I8.Body stability skills	Agreement	17 (85)	12 (60)	YES
I9.Physical activity	Agreement	19 (95.5)	14 (70)	YES
I10.Motor behavior	Agreement	18 (90)	10 (50)	YES
I11.Emotional mastery	Agreement	18 (90)	12 (60)	YES
I12.self-recognition	Agreement	18 (90)	10 (50)	YES
I13.Dare to challenge	Agreement	17 (85)	14 (70)	YES
I14.Friendly competition	Agreement	19 (95.5)	9 (45)	YES
I15.Respect for order	Agreement	18 (90)	16 (80)	YES
I16.Self-protection	Agreement	19 (95.5)	17 (85)	YES
I17.Willingness to cooperate	Agreement	17 (85)	7 (35)	YES
Contents (C)				
C1.Hand-eye coordination	Agreement	19 (95.5)	16 (80)	YES
C2.Hand-foot coordination	Agreement	19 (95.5)	12 (60)	YES
C3.Reaction velocity	Agreement	19 (95.5)	8 (40)	YES
C4.Displacement velocity	Disagreement	15 (75)	9 (45)	YES
C5.Dynamic balance	Agreement	18 (90)	13 (65)	YES
C6.Static balance	Agreement	17 (85)	10 (50)	YES
C7.Upper body Strength	Agreement	17 (85)	7 (35)	YES
C8.Lower body strength	Agreement	17 (85)	7 (35)	YES
C9.Cardiorespiratory endurance	Agreement	18 (90)	8 (40)	YES
C10.Walk	Agreement	19 (95.5)	17 (85)	YES
C11.Run	Agreement	19 (95.5)	17 (85)	YES
C12.Skip	Agreement	19 (95.5)	17 (85)	YES
C13.Climb	Agreement	18 (90)	14 (70)	YES
C14.Straddle	Agreement	17 (85)	11 (55)	YES
C15.Slide	Agreement	17 (85)	5 (25)	YES
C16.Racket the ball	Agreement	18 (90)	12 (60)	YES
C17.Hit the ball	Agreement	18 (90)	8 (40)	YES
C18.Passing and receiving the ball	Agreement	18 (90)	9 (45)	YES
C19.Throwing the ball	Agreement	18 (90)	10 (50)	YES
C20.Kick the ball	Agreement	17 (85)	9 (45)	YES
C21.Roll	Agreement	19 (95.5)	9 (45)	YES
C22.Whirl	Agreement	18 (90)	8 (40)	YES
C23.Hedge	Agreement	18 (90)	8 (40)	YES
C24.Motor instruction	Agreement	18 (90)	9 (45)	YES
C25.Music rhythm	Agreement	19 (95.5)	12 (60)	YES

## 4 Discussion

This study developed a system of LEG curriculum indicators using an interdisciplinary approach to foster the healthy and sustainable development of preschoolers aged 3–6 years. This initiative seeks to address public health challenges associated with inadequate levels of physical activity and delays in fundamental movement skills. The LEG curriculum indicator system has demonstrated considerable consistency and stability following three rounds of surveys, which included three objectives, seven tasks, 17 indicators, and 25 content areas. In the initial round of the survey, the following indicators were not met (i.e., registered below 75%): T5 (competitive awareness), I5 (quality of endurance), I14 (dare to take risks), C12 (muscular endurance), C18 (slide), and C22 (throw the ball). Regarding competitive awareness(T5), kinesiology research has determined that sports participation involves a process of competition and rivalry (23). Moreover, it is evident that participation in sports-related activities not only reinforce motor skills and support social development (24) but also aid in the emotional and behavioral regulation of children (25), establishing it as an optimal educational approach. However, existing research on preschool settings indicates that the outcomes derived from competitions and contests in young children’s play may inadvertently cause psychological harm to vulnerable children.

The analysis concluded that the lack of competition and challenges in outdoor activities make it difficult for young children to achieve moderate, intermediate-high, and high activity levels. Additionally, the emphasis on sports safety and the use of free activities, along with a semi-structured curriculum for outdoor programs, may result in low levels of physical activity and hinder the development of basic motor skills, which aligns with the research of Rosita et al. (26–28).

Regarding the quality of endurance (I5) and muscular endurance (C12), the I5 standard was not met in the first and second rounds of the survey (<75%). This result arises from cognitive differences in the discipline and from research in kinesiology, which considers the quality of endurance a critical component of physical capacity. National studies on the physical fitness of young children emphasize promoting physical endurance to support children’s health (3, 14, 29, 30). Proper development of muscular endurance in young children can enhance the quality of physical activity. However, preschool pedagogical studies have concluded that young children’s physiological development is still incomplete; they are susceptible to fatigue, and developing endurance requires prolonged participation in physical activity, which may lead to safety accidents in sports (31). Therefore, this study acknowledged the physical development patterns of preschool children aged 3–6 years by excluding the muscular endurance indicator (C12) while retaining the endurance (I5) and cardiorespiratory endurance (C11) indicators in the results of the second research round, guaranteeing that children aged 1–4 years require at least 180 min of physical activity of varying intensity per day, while children aged 3–4 years need a minimum of 60 min of moderate to vigorous physical activity.

Regarding the dare to engage in risk-taking (I14), experts in preschool pedagogy assert that young children are incapable of independently achieving self-protection when confronted with hazardous situations. They argue that incorporating risk-taking content into the curriculum may misguide young children’s judgment regarding dangerous scenarios and potentially result in physical and psychological harm due to sports-related accidents; consequently, this indicator has been omitted. In relation to the activities of sliding (C18) and throwing a ball (C22), these activities demonstrate strong reliability and validity across various national assessments as indicators within the Test of Gross Motor Development (TGMD) scale for children aged 3–10 years [32–35]. A consensus for their retention was achieved during the second round of the survey following consultations with experts. The indicators pertaining to the speed of body movement (C5) and lumbar and abdominal strength (C9) have not been thoroughly examined in studies focused on large muscle movements among children aged 3–6 years. They present greater challenges in realization and assessment in relation to the development of large muscle movements in young children. Moreover, the C5 indicator is largely dependent on the degree of innervation of the nervous system relative to the muscles, which poses difficulties in quantification. As a result, indicators C5 and C9 were eliminated in the third round of the Delphi survey. Indicators I2, I4, C4, C8, and C19 have demonstrated relevance in international studies concerning the physical development of young children, thus leading to the decision to retain these five indicators for the third round of the survey (30, 32–36).

After three rounds of Delphi research, the LEG curriculum objectives and content indicator system consisted of three objectives, seven tasks, 17 indicators, and 25 curricular contents. They are essential for promoting the sustainable enhancement of FMS and PA in preschool children aged 3–6 years.

## 5 Conclusion

The findings of this study suggest that discussing and reaching a consensus from an applied research perspective in kinesiology and preschool education is a complex and meaningful endeavor. This Delphi research consensus may have led to the development of a new model for early childhood physical education curriculum that effectively addresses the lack of physical activity and the delayed development of fundamental movement skills in young children. It also lays a foundation for future empirical research on LEG curricula. The study clearly indicates that there are distinct disciplinary backgrounds and geographic and cultural differences within the interdisciplinary applied research of kinesiology and preschool education. When confronted with such differences, we chose to respect the laws of physical development of preschoolers aged 3–6 years and prioritized safety. We believe that these differences can be gradually narrowed or replaced as applied research progresses. Future studies on outdoor physical activity programs for young children are likely to favor a highly structured curriculum model. The learning movements, exercise movements, and game activities included in this more structured curriculum may be key to achieving sustainable improvements in fundamental movement skills and physical activity among preschool children aged 3–6 years.

## 6 Limitations

Although the LEG program indicator system aims to promote the healthy and sustainable development of preschool children aged 3–6 years, it has only reached a consensus on three objectives, seven tasks, 17 indicators, and 25 curricular contents. This merely serves as a justification of the LEG program’s objectives and contents, highlighting a notable lack of empirical research concerning the effects of physical abilities, health behaviors, and motor cognition. In the future, we will emphasize researching the impact of LEG programs on preschool children’s physical activity levels and basic motor skills, further substantiating the idea that LEG programs contribute to the ongoing development of preschool children’s physical activity and fundamental movement skills.

## Data Availability

The original contributions presented in the study are included in the article/Supplementary material, further inquiries can be directed to the corresponding author/s.
