# Supplementary material for: The LEG program promotes the development of physical activity and fundamental movement skills in preschool children aged 3–6 years: a Delphi study
Source: Front Public Health. 2025 Mar 25;13:1521878. doi: 10.3389/fpubh.2025.1521878 (PMC11975950; doi:10.3389/fpubh.2025.1521878)
Supplement: Supplementary file 1 [file Data_Sheet_1.docx]

Questionnaire on LEG Course Indicator System (Round one)

**Dear Experts:**

We are trying to construct a LEG curriculum indicator system from an interdisciplinary perspective of kinesiology and preschool education, and we are using the Delphi method to investigate and justify it.LEG is a structured curriculum designed for preschool children aged 3-6 years old based on the optimization of the curriculum model of teaching, practicing and competing in youth sports, in order to achieve the goal of "enjoying fun, strengthening physical fitness, improving personality and refining will", with the aim of promoting the simultaneous development of physical activity and basic motor skills of preschool children aged 3-6 years old. The purpose of the program is to promote the synchronized development of physical activity and fundamental movement skills of preschool children aged 3-6.It consists of 5 sessions: Preparatory activities, learning movement (L), exercising movement (E), Game activities (G) and Relaxation activity sessions. Please rate its importance according to your education and teaching experience, with your participation we can get reliable conclusions, thank you for participating in the survey in your busy schedule.

June 03, 2024

**Part I Basic Information**

**1. Your gender?**

○ Male

○ Female

**2.What is your education level?**

○ Bachelor's degree

○ Master's degree

○ Doctoral degree

**3.What is the nature of your work organization?**

○ Research organization(College)

○ Early education organization

○ Education and training institutions

**4.What is your area of specialization?**

○ Kinesiology

○ Pre-primary education

**5.How many years have you been in your area of specialization?**

○ 1-5 years

○ 6~10 years

○ 11~15 years

○ More than 15 years

**Part II Description of the questionnaire**

(1) This questionnaire sets up 3 objectives, 8 tasks , 20 indicators and 29 contents. Please refer to the whole indicator system and evaluate the importance of indicators at all levels.

(2) The evaluation score is from 1 to 5, with a minimum of 1 and a maximum of 5. The higher the score, the more you agree with it, so please rate it according to your experience.

(3) If there are any omissions in this questionnaire, please add them in "New Indicators" and "Expert Advice".

Table 1 shows the LEG Curriculum Objectives and Content Indicators (Round 1), which we will combine with expert scoring to determine the indicator system for subsequent scoring and indicator adjustment.

Table 1: LEG Curriculum Objectives and Content Indicators (Round 1)

| **Objectives (O)** | **Tasks (T)** | **Indicators (I)** | **Content s(C)** |
| --- | --- | --- | --- |
| O1.Physical capability | T1.Physical fitness | I1.Body coordination | C1.Hand-eye coordination  C2.Hand-foot coordination  C3.Reaction velocity  C4.Displacement velocity  C5.Velocity of body movement  C6.Dynamic balance  C7.Static balance  C8.Upper body Strength  C9.Lumbar and abdominal strength  C10.Lower body strength  C11.Cardiorespiratory endurance  C12.Muscle endurance  C13.Walk  C14.Run  C15.Skip  C16.Climb  C17.Straddle  C18.Slide  C19.Racket the ball  C20.Hit the ball  C21.Passing and receiving the ball  C22.Throw the ball  C23.Throwing a ball  C24.Kick the ball  C25.Roll  C26.Whirl  C27.Leap  C28.Hedge |
|  |  | I2.Quality of velocity |  |
|  |  | I3.Balance |  |
|  |  | I4.Quality of strength |  |
|  |  | I5.Quality of endurance |  |
|  | T2.Motor skills | I6.Body movement skills |  |
|  |  | I7.Object control skills |  |
|  |  | I8.Body stability skills |  |
| O2.Healthy behaviors | T3.Body health | I9.Physical activity |  |
|  |  | I10.Motor behavior |  |
|  | T4.Psychological health | I11.Emotional mastery |  |
|  |  | I12.Pro-social behavior |  |
| O3.Motor cognition | T5.Competitive awareness | I13.Courage to challenge |  |
|  |  | I14.Dare to take risks |  |
|  | T6.Rule awareness | I15.Respect for order |  |
|  |  | I16.Respect for discipline |  |
|  | T7.Safety awareness | I17.Self-protection |  |
|  |  | I18.Sense of responsibility |  |
|  | T8.Teamwork awareness | I19.Teamwork spirit |  |
|  |  | I20.Willingness to cooperate |  |

Table 2 represents the scale of importance of the indicators. There are three levels of the course indicator system, so please rate the importance of the previous level of indicators corresponding to each level of indicators based on your experience.

Table 2 the scale of importance of the indicators**.**

| **Importance** | **Not important** | **Not very important** | **General important** | **Important** | **Very important** |
| --- | --- | --- | --- | --- | --- |
| **Score** | **1** | **2** | **3** | **4** | **5** |

**Part III Indicator scorecard**

| **Items/Score** | **1** | **2** | **3** | **4** | **5** |
| --- | --- | --- | --- | --- | --- |
| O1.Physical capability | ○ | ○ | ○ | ○ | ○ |
| O2.Healthy behaviors | ○ | ○ | ○ | ○ | ○ |
| O3.Motor cognition | ○ | ○ | ○ | ○ | ○ |
| **Do you suggest a new indicator? Fill in the name of the indicator: ______________________， and fill in the indicator score ____.** | | | | | |

**O1.Physical capability**

| **Items/Score** | **1** | **2** | **3** | **4** | **5** |
| --- | --- | --- | --- | --- | --- |
| T1.Physical fitness | ○ | ○ | ○ | ○ | ○ |
| T2.Motor skills | ○ | ○ | ○ | ○ | ○ |
| **Do you suggest a new indicator? Fill in the name of the indicator: ______________________， and fill in the indicator score ____.** | | | | | |

**O2.Healthy behaviors**

| **Items/Score** | **1** | **2** | **3** | **4** | **5** |
| --- | --- | --- | --- | --- | --- |
| T3.Body health | ○ | ○ | ○ | ○ | ○ |
| T4.Psychological health | ○ | ○ | ○ | ○ | ○ |
| **Do you suggest a new indicator? Fill in the name of the indicator: ______________________， and fill in the indicator score ____.** | | | | | |

**O3.Motor cognition**

| **Items/Score** | **1** | **2** | **3** | **4** | **5** |
| --- | --- | --- | --- | --- | --- |
| T5.Competitive awareness | ○ | ○ | ○ | ○ | ○ |
| T6.Rule awareness | ○ | ○ | ○ | ○ | ○ |
| T7.Safety awareness | ○ | ○ | ○ | ○ | ○ |
| T8.Teamwork awareness | ○ | ○ | ○ | ○ | ○ |
| **Do you suggest a new indicator? Fill in the name of the indicator: ______________________， and fill in the indicator score ____.** | | | | | |

**T1.Physical fitness**

| **Items/Score** | **1** | **2** | **3** | **4** | **5** |
| --- | --- | --- | --- | --- | --- |
| I1.Body coordination | ○ | ○ | ○ | ○ | ○ |
| I2.Quality of velocity | ○ | ○ | ○ | ○ | ○ |
| I3.Balance | ○ | ○ | ○ | ○ | ○ |
| I4.Quality of strength | ○ | ○ | ○ | ○ | ○ |
| I5.Quality of endurance | ○ | ○ | ○ | ○ | ○ |
| **Do you suggest a new indicator? Fill in the name of the indicator: ______________________， and fill in the indicator score ____.** | | | | | |

**T2.Motor skills**

| **Items/Score** | **1** | **2** | **3** | **4** | **5** |
| --- | --- | --- | --- | --- | --- |
| I6.Body movement skills | ○ | ○ | ○ | ○ | ○ |
| I7.Object control skills | ○ | ○ | ○ | ○ | ○ |
| I8.Body stability skills | ○ | ○ | ○ | ○ | ○ |
| **Do you suggest a new indicator? Fill in the name of the indicator: ______________________， and fill in the indicator score ____.** | | | | | |

**T3.Body health**

| **Items/Score** | **1** | **2** | **3** | **4** | **5** |
| --- | --- | --- | --- | --- | --- |
| I9.Physical activity | ○ | ○ | ○ | ○ | ○ |
| I10.Motor behavior | ○ | ○ | ○ | ○ | ○ |
| **Do you suggest a new indicator? Fill in the name of the indicator: ______________________， and fill in the indicator score ____.** | | | | | |

**T4.Psychological health**

| **Items/Score** | **1** | **2** | **3** | **4** | **5** |
| --- | --- | --- | --- | --- | --- |
| I11.Emotional mastery | **○** | **○** | **○** | **○** | **○** |
| I12.Pro-social behavior | **○** | **○** | **○** | **○** | **○** |
| **Do you suggest a new indicator? Fill in the name of the indicator: ______________________， and fill in the indicator score ____.** | | | | | |

**T5.Competitive awareness**

| **Items/Score** | **1** | **2** | **3** | **4** | **5** |
| --- | --- | --- | --- | --- | --- |
| I13.Courage to challenge | ○ | ○ | ○ | ○ | ○ |
| I14.Dare to take risks | ○ | ○ | ○ | ○ | ○ |
| **Do you suggest a new indicator? Fill in the name of the indicator: ______________________， and fill in the indicator score ____.** | | | | | |

**T6.Rule awareness**

| **Items/Score** | **1** | **2** | **3** | **4** | **5** |
| --- | --- | --- | --- | --- | --- |
| I15.Respect for order | ○ | ○ | ○ | ○ | ○ |
| I16.Respect for discipline | ○ | ○ | ○ | ○ | ○ |
| **Do you suggest a new indicator? Fill in the name of the indicator: ______________________， and fill in the indicator score ____.** | | | | | |

**T7.Safety awareness**

| **Items/Score** | **1** | **2** | **3** | **4** | **5** |
| --- | --- | --- | --- | --- | --- |
| I17.Self-protection | ○ | ○ | ○ | ○ | ○ |
| I18.Sense of responsibility | ○ | ○ | ○ | ○ | ○ |
| **Do you suggest a new indicator? Fill in the name of the indicator: ______________________， and fill in the indicator score ____.** | | | | | |

**T8.Teamwork awareness**

| **Items/Score** | **1** | **2** | **3** | **4** | **5** |
| --- | --- | --- | --- | --- | --- |
| I19.Teamwork spirit | ○ | ○ | ○ | ○ | ○ |
| I20.Willingness to cooperate | ○ | ○ | ○ | ○ | ○ |
| **Do you suggest a new indicator? Fill in the name of the indicator: ______________________， and fill in the indicator score ____.** | | | | | |

**LEG course content indicators**

| **Items/Score** | **1** | **2** | **3** | **4** | **5** |
| --- | --- | --- | --- | --- | --- |
| C1.Hand-eye coordination | ○ | ○ | ○ | ○ | ○ |
| C2.Hand-foot coordination | ○ | ○ | ○ | ○ | ○ |
| C3.Reaction velocity | ○ | ○ | ○ | ○ | ○ |
| C4.Displacement velocity | ○ | ○ | ○ | ○ | ○ |
| C5.Velocity of body movement | ○ | ○ | ○ | ○ | ○ |
| C6.Dynamic balance | ○ | ○ | ○ | ○ | ○ |
| C7.Static balance | ○ | ○ | ○ | ○ | ○ |
| C8.Upper body Strength | ○ | ○ | ○ | ○ | ○ |
| C9.Lumbar and abdominal strength | ○ | ○ | ○ | ○ | ○ |
| C10.Lower body strength | ○ | ○ | ○ | ○ | ○ |
| C11.Cardiorespiratory endurance | ○ | ○ | ○ | ○ | ○ |
| C12.Muscle endurance | ○ | ○ | ○ | ○ | ○ |
| C13.Walk | ○ | ○ | ○ | ○ | ○ |
| C14.Run | ○ | ○ | ○ | ○ | ○ |
| C15.Skip | ○ | ○ | ○ | ○ | ○ |
| C16.Climb | ○ | ○ | ○ | ○ | ○ |
| C17.Straddle | ○ | ○ | ○ | ○ | ○ |
| C18.Slide | ○ | ○ | ○ | ○ | ○ |
| C19.Racket the ball | ○ | ○ | ○ | ○ | ○ |
| C20.Hit the ball | ○ | ○ | ○ | ○ | ○ |
| C21.Passing and receiving the ball | ○ | ○ | ○ | ○ | ○ |
| C22.Throw the ball | ○ | ○ | ○ | ○ | ○ |
| C23.Throwing a ball | ○ | ○ | ○ | ○ | ○ |
| C24.Kick the ball | ○ | ○ | ○ | ○ | ○ |
| C25.Roll | ○ | ○ | ○ | ○ | ○ |
| C26.Whirl | ○ | ○ | ○ | ○ | ○ |
| C27.Leap | ○ | ○ | ○ | ○ | ○ |
| C28.Hedge | ○ | ○ | ○ | ○ | ○ |
| **Do you suggest a new indicator? Fill in the name of the indicator: ______________________， and fill in the indicator score ____.** | | | | | |

| Do you have any other suggestions for the LEG indicator system? Suggested: ________________________ ，fill in the indicator score ____. |
| --- |

**Thank you very much for your help and I wish you all the best in your work and life!**
