# Supplementary material for: The LEG program promotes the development of physical activity and fundamental movement skills in preschool children aged 3–6 years: a Delphi study
Source: Front Public Health. 2025 Mar 25;13:1521878. doi: 10.3389/fpubh.2025.1521878 (PMC11975950; doi:10.3389/fpubh.2025.1521878)
Supplement: Supplementary file 2 [file Data_Sheet_2.docx]

Questionnaire on LEG Course Indicator System (Round two)

**Dear Experts:**

We are trying to construct a LEG curriculum indicator system from an interdisciplinary perspective of Kinesiology and Preschool education, and we are using the Delphi method to investigate and justify it.After the first round of surveys and interviews, the research on this project has made good progress, but consensus could not be well reached on some indicators. In this round of survey, we have added, deleted, modified and merged some indicators according to the experts' opinions in the first round. Please fill in the survey again according to your education and teaching experience. Thank you for participating in the survey in your busy schedule.

June 23, 2024

**Part I Basic Information**

**1. Your gender?**

○ Male

○ Female

**2.What is your education level?**

○ Bachelor's degree

○ Master's degree

○ Doctoral degree

**3.What is the nature of your work organization?**

○ Research organization(College)

○ Early education organization

○ Education and training institutions

**4.What is your area of specialization?**

○ Kinesiology

○ Pre-primary education

**5.How many years have you been in your area of specialization?**

○ 1-5 years

○ 6~10 years

○ 11~15 years

○ More than 15 years

**Part II Description of the questionnaire**

(1) This questionnaire sets up 3 objectives, 7 tasks, 17indicators and 27contents. Please refer to the whole indicator system and evaluate the importance of indicators at all levels.

(2) The evaluation score is from 1 to 5, with a minimum of 1 and a maximum of 5. The higher the score, the more you agree with it, so please rate it according to your experience.

(3) If there are any omissions in this questionnaire, please add them in "New Indicators" and "Expert Advice".

Table 1 shows the LEG Curriculum Objectives and Content Indicators (Round 2), which we will combine with expert scoring to determine the indicator system for subsequent scoring and indicator adjustment.

Table 1: LEG Curriculum Objectives and Content Indicators (Round 2)

| **Objectives (O)** | **Tasks (T)** | **Indicators (I)** | **Contents (C)** |
| --- | --- | --- | --- |
| O1.Physical capability | T1:Physical fitness | I1.Body coordination | C1.Hand-eye coordination  C2.Hand-foot coordination  C3.Reaction velocity  C4.Displacement velocity  C5.Velocity of body movement  C6.Dynamic balance  C7.Static balance  C8.Upper body Strength  C9.Lumbar and abdominal strength  C10.Lower body strength  C11.Cardiorespiratory endurance  C12.Walk  C13.Run  C14.Skip  C15.Climb  C16.Straddle  C17.Slide  C18.Racket the ball  C19.Hit the ball  C20.Passing and receiving the ball  C21.Throwing the ball  C22.Kick the ball  C23.Roll  C24.Whirl  C25.Hedge  C26.Movement instruction  C27.Music rhythm. |
|  |  | I2.Quality of velocity |  |
|  |  | I3.Balance |  |
|  |  | I4.Quality of strength |  |
|  |  | I5.Quality of endurance |  |
|  | T2：Motor skills | I6.Body movement skills |  |
|  |  | I7.Object control skills |  |
|  |  | I8.Body stability skills |  |
| O2.Healthy behaviors | T3:Body health | I9.Physical activity |  |
|  |  | I10.Motor behavior |  |
|  | T4:Psychological health | I11.Emotional mastery |  |
|  |  | I12.self-recognition |  |
| O3.Motor cognition | T5:Rule awareness | I13.Dare to challenge |  |
|  |  | I14.Friendly competition |  |
|  |  | I15.Respect for order |  |
|  | T6:Safety awareness | I16.Self-protection |  |
|  | T7:Teamwork awareness | I17.Willingness to cooperate |  |

Table 2 represents the scale of importance of the indicators. There are three levels of the course indicator system, so please rate the importance of the previous level of indicators corresponding to each level of indicators based on your experience.

Table 2 the scale of importance of the indicators**.**

| **Importance** | **Not important** | **Not very important** | **General important** | **Important** | **Very important** |
| --- | --- | --- | --- | --- | --- |
| **Score** | **1** | **2** | **3** | **4** | **5** |

**Part III Indicator scorecard**

| **Items/Score** | **1** | **2** | **3** | **4** | **5** |
| --- | --- | --- | --- | --- | --- |
| O1.Physical capability | ○ | ○ | ○ | ○ | ○ |
| O2.Healthy behaviors | ○ | ○ | ○ | ○ | ○ |
| O3.Motor cognition | ○ | ○ | ○ | ○ | ○ |
| **Do you suggest a new indicator? Fill in the name of the indicator: ______________________， and fill in the indicator score ____.** | | | | | |

**O1.Physical capability**

| **Items/Score** | **1** | **2** | **3** | **4** | **5** |
| --- | --- | --- | --- | --- | --- |
| T1.Physical fitness | ○ | ○ | ○ | ○ | ○ |
| T2.Motor skills | ○ | ○ | ○ | ○ | ○ |
| **Do you suggest a new indicator? Fill in the name of the indicator: ______________________， and fill in the indicator score ____.** | | | | | |

**O2.Healthy behaviors**

| **Items/Score** | **1** | **2** | **3** | **4** | **5** |
| --- | --- | --- | --- | --- | --- |
| T3.Body health | ○ | ○ | ○ | ○ | ○ |
| T4.Psychological health | ○ | ○ | ○ | ○ | ○ |
| **Do you suggest a new indicator? Fill in the name of the indicator: ______________________， and fill in the indicator score ____.** | | | | | |

**O3.Motor cognition**

| **Items/Score** | **1** | **2** | **3** | **4** | **5** |
| --- | --- | --- | --- | --- | --- |
| T5.Rule awareness | ○ | ○ | ○ | ○ | ○ |
| T6.Safety awareness | ○ | ○ | ○ | ○ | ○ |
| T7.Teamwork awareness | ○ | ○ | ○ | ○ | ○ |
| **Do you suggest a new indicator? Fill in the name of the indicator: ______________________， and fill in the indicator score ____.** | | | | | |

**T1.Physical fitness**

| **Items/Score** | **1** | **2** | **3** | **4** | **5** |
| --- | --- | --- | --- | --- | --- |
| I1.Body coordination | ○ | ○ | ○ | ○ | ○ |
| I2.Quality of velocity | ○ | ○ | ○ | ○ | ○ |
| I3.Balance | ○ | ○ | ○ | ○ | ○ |
| I4.Quality of strength | ○ | ○ | ○ | ○ | ○ |
| I5.Quality of endurance | ○ | ○ | ○ | ○ | ○ |
| **Do you suggest a new indicator? Fill in the name of the indicator: ______________________， and fill in the indicator score ____.** | | | | | |

**T2.Motor skills**

| **Items/Score** | **1** | **2** | **3** | **4** | **5** |
| --- | --- | --- | --- | --- | --- |
| I6.Body movement skills | ○ | ○ | ○ | ○ | ○ |
| I7.Object control skills | ○ | ○ | ○ | ○ | ○ |
| I8.Body stability skills | ○ | ○ | ○ | ○ | ○ |
| **Do you suggest a new indicator? Fill in the name of the indicator: ______________________， and fill in the indicator score ____.** | | | | | |

**T3.Body health**

| **Items/Score** | **1** | **2** | **3** | **4** | **5** |
| --- | --- | --- | --- | --- | --- |
| I9.Physical activity | ○ | ○ | ○ | ○ | ○ |
| I10.Motor behavior | ○ | ○ | ○ | ○ | ○ |
| **Do you suggest a new indicator? Fill in the name of the indicator: ______________________， and fill in the indicator score ____.** | | | | | |

**T4.Psychological health**

| **Items/Score** | **1** | **2** | **3** | **4** | **5** |
| --- | --- | --- | --- | --- | --- |
| I11.Emotional mastery | **○** | **○** | **○** | **○** | **○** |
| I12.self-recognition | **○** | **○** | **○** | **○** | **○** |
| **Do you suggest a new indicator? Fill in the name of the indicator: ______________________， and fill in the indicator score ____.** | | | | | |

**T5.Rule awareness**

| **Items/Score** | **1** | **2** | **3** | **4** | **5** |
| --- | --- | --- | --- | --- | --- |
| I13.Dare to challenge | ○ | ○ | ○ | ○ | ○ |
| I14.Friendly competition | ○ | ○ | ○ | ○ | ○ |
| I15.Respect for order | ○ | ○ | ○ | ○ | ○ |
| **Do you suggest a new indicator? Fill in the name of the indicator: ______________________， and fill in the indicator score ____.** | | | | | |

**T6.Safety awareness**

| **Items/Score** | **1** | **2** | **3** | **4** | **5** |
| --- | --- | --- | --- | --- | --- |
| I16.Self-protection | ○ | ○ | ○ | ○ | ○ |
| **Do you suggest a new indicator? Fill in the name of the indicator: ______________________， and fill in the indicator score ____.** | | | | | |

**T7.Teamwork awareness**

| **Items/Score** | **1** | **2** | **3** | **4** | **5** |
| --- | --- | --- | --- | --- | --- |
| I17.Willingness to cooperate | ○ | ○ | ○ | ○ | ○ |
| **Do you suggest a new indicator? Fill in the name of the indicator: ______________________， and fill in the indicator score ____.** | | | | | |

**LEG course content indicators**

| **Items/Score** | **1** | **2** | **3** | **4** | **5** |
| --- | --- | --- | --- | --- | --- |
| C1.Hand-eye coordination | ○ | ○ | ○ | ○ | ○ |
| C2.Hand-foot coordination | ○ | ○ | ○ | ○ | ○ |
| C3.Reaction velocity | ○ | ○ | ○ | ○ | ○ |
| C4.Displacement velocity | ○ | ○ | ○ | ○ | ○ |
| C5.Velocity of body movement | ○ | ○ | ○ | ○ | ○ |
| C6.Dynamic balance | ○ | ○ | ○ | ○ | ○ |
| C7.Static balance | ○ | ○ | ○ | ○ | ○ |
| C8.Upper body Strength | ○ | ○ | ○ | ○ | ○ |
| C9.Lumbar and abdominal strength | ○ | ○ | ○ | ○ | ○ |
| C10.Lower body strength | ○ | ○ | ○ | ○ | ○ |
| C11.Cardiorespiratory endurance | ○ | ○ | ○ | ○ | ○ |
| C12.Walk | ○ | ○ | ○ | ○ | ○ |
| C13.Run | ○ | ○ | ○ | ○ | ○ |
| C14.Skip | ○ | ○ | ○ | ○ | ○ |
| C15.Climb | ○ | ○ | ○ | ○ | ○ |
| C16.Straddle | ○ | ○ | ○ | ○ | ○ |
| C17.Slide | ○ | ○ | ○ | ○ | ○ |
| C18.Racket the ball | ○ | ○ | ○ | ○ | ○ |
| C19.Hit the ball | ○ | ○ | ○ | ○ | ○ |
| C20.Passing and receiving the ball | ○ | ○ | ○ | ○ | ○ |
| C21.Throwing the ball | ○ | ○ | ○ | ○ | ○ |
| C22.Kick the ball | ○ | ○ | ○ | ○ | ○ |
| C23.Roll | ○ | ○ | ○ | ○ | ○ |
| C24.Whirl | ○ | ○ | ○ | ○ | ○ |
| C25.Hedge | ○ | ○ | ○ | ○ | ○ |
| C26.Movement instruction | ○ | ○ | ○ | ○ | ○ |
| C27.Music rhythm | ○ | ○ | ○ | ○ | ○ |
| **Do you suggest a new indicator? Fill in the name of the indicator: ______________________， and fill in the indicator score ____.** | | | | | |

| Do you have any other suggestions for the LEG indicator system? Suggested: ________________________ ，fill in the indicator score ____. |
| --- |

**Thank you very much for your help and I wish you all the best in your work and life!**
