# Supplementary material for: The LEG program promotes the development of physical activity and fundamental movement skills in preschool children aged 3–6 years: a Delphi study
Source: Front Public Health. 2025 Mar 25;13:1521878. doi: 10.3389/fpubh.2025.1521878 (PMC11975950; doi:10.3389/fpubh.2025.1521878)
Supplement: Supplementary file 4 [file Table_1.docx]

**Results of Round One of the Delphi Study**

| **Items** | **Median** | **Mean** | **SD** | **IQD** |
| --- | --- | --- | --- | --- |
| **Objectives (O)** |  |  |  |  |
| O1.Physical capability | 5.00 | 4.64 | 0.581 | 1.000 |
| O2.Healthy behaviors | 5.00 | 4.50 | 0.598 | 1.000 |
| O3.Motor cognition | 4.00 | 4.18 | 0.795 | 1.000 |
| **Tasks (T)** |  |  |  |  |
| T1.Physical fitness | 5.00 | 4.55 | 0.739 | 1.000 |
| T2.Motor skills | 4.50 | 4.32 | 0.780 | 1.000 |
| T3.Body health | 5.00 | 4.64 | 0.658 | 1.000 |
| T4.Psychological health | 5.00 | 4.55 | 0.739 | 1.000 |
| T5.Competitive awareness | 4.00 | 3.91 | 1.019 | 2.000 |
| T6.Rule awareness | 5.00 | 4.55 | 0.671 | 1.000 |
| T7.Safety awareness | 5.00 | 4.73 | 0.550 | 0.250 |
| T8.Teamwork awareness | 5.00 | 4.64 | 0.581 | 1.000 |
| **Indicators (I)** |  |  |  |  |
| I1.Body coordination | 5.00 | 4.68 | 0.646 | 0.250 |
| I2.Quality of velocity | 4.00 | 4.18 | 0.907 | 1.250 |
| I3.Balance | 5.00 | 4.59 | 0.666 | 1.000 |
| I4.Quality of strength | 4.00 | 4.18 | 0.795 | 1.250 |
| I5.Quality of endurance | 4.00 | 4.00 | 1.024 | 2.000 |
| I6.Body movement skills | 5.00 | 4.68 | 0.568 | 1.000 |
| I7.Object control skills | 5.00 | 4.59 | 0.590 | 1.000 |
| I8.Body stability skills | 5.00 | 4.50 | 0.740 | 1.000 |
| I9.Physical activity | 5.00 | 4.45 | 0.671 | 1.000 |
| I10.Motor behavior | 5.00 | 4.41 | 0.734 | 1.000 |
| I11.Emotional mastery | 5.00 | 4.55 | 0.739 | 1.000 |
| I12.Pro-social behavior | 5.00 | 4.32 | 0.945 | 1.000 |
| I13.Courage to challenge | 5.00 | 4.50 | 0.802 | 1.000 |
| I14.Dare to take risks | 4.00 | 4.09 | 0.971 | 2.000 |
| I15.Respect for order | 5.00 | 4.82 | 0.395 | 0.000 |
| I16.Respect for discipline | 5.00 | 4.55 | 0.671 | 1.000 |
| I17.Self-protection | 5.00 | 4.86 | 0.351 | 0.000 |
| I18.Sense of responsibility | 4.00 | 4.27 | 0.767 | 1.000 |
| I19.Teamwork spirit | 5.00 | 4.50 | 0.598 | 1.000 |
| I20.Willingness to cooperate | 5.00 | 4.68 | 0.477 | 1.000 |
| **Contents (C)** |  |  |  |  |
| C1.Hand-eye coordination | 5.00 | 4.64 | 0.581 | 1.000 |
| C2.Hand-foot coordination | 5.00 | 4.55 | 0.671 | 1.000 |
| C3.Reaction velocity | 5.00 | 4.41 | 0.734 | 1.000 |
| C4.Displacement velocity | 4.00 | 4.23 | 0.685 | 1.000 |
| C5.Velocity of body movement | 4.00 | 4.36 | 0.658 | 1.000 |
| C6.Dynamic balance | 5.00 | 4.64 | 0.581 | 1.000 |
| C7.Static balance | 5.00 | 4.41 | 0.734 | 1.000 |
| C8.Upper body Strength | 4.00 | 4.14 | 0.889 | 1.250 |
| C9.Lumbar and abdominal strength | 5.00 | 4.45 | 0.671 | 1.000 |
| C10.Lower body strength | 4.50 | 4.36 | 0.727 | 1.000 |
| C11.Cardiorespiratory endurance | 4.00 | 4.23 | 0.813 | 1.250 |
| C12.Muscle endurance | 4.00 | 4.00 | 0.926 | 2.000 |
| C13.Walk | 4.50 | 4.45 | 0.596 | 1.000 |
| C14.Run | 5.00 | 4.64 | 0.581 | 1.000 |
| C15.Skip | 5.00 | 4.55 | 0.739 | 1.000 |
| C16.Climb | 5.00 | 4.50 | 0.673 | 1.000 |
| C17.Straddle | 4.00 | 4.36 | 0.581 | 1.000 |
| C18.Slide | 4.00 | 4.00 | 0.873 | 1.000 |
| C19.Racket the ball | 4.00 | 4.36 | 0.581 | 1.000 |
| C20.Hit the ball | 4.00 | 4.09 | 0.868 | 1.250 |
| C21.Passing and receiving the ball | 4.00 | 4.00 | 0.690 | 0.500 |
| C22.Throw the ball | 4.00 | 4.09 | 0.811 | 2.000 |
| C23.Throwing a ball | 4.00 | 4.05 | 0.722 | 1.250 |
| C24.Kick the ball | 4.00 | 4.18 | 0.733 | 1.000 |
| C25.Roll | 4.00 | 4.23 | 0.813 | 1.250 |
| C26.Whirl | 4.00 | 4.23 | 0.813 | 1.250 |
| C27.Leap | 5.00 | 4.45 | 0.671 | 1.000 |
| C28.Hedge | 5.00 | 4.59 | 0.503 | 1.000 |
