# Supplementary material for: The LEG program promotes the development of physical activity and fundamental movement skills in preschool children aged 3–6 years: a Delphi study
Source: Front Public Health. 2025 Mar 25;13:1521878. doi: 10.3389/fpubh.2025.1521878 (PMC11975950; doi:10.3389/fpubh.2025.1521878)
Supplement: Supplementary file 5 [file Table_2.doc]

**Results of Round Two of the Delphi Study**

| **Items** | **Median** | **Mean** | **SD** | **IQD** |
| --- | --- | --- | --- | --- |
| **Objectives (O)** |  |  |  |  |
| O1.Physical capability | 5.00 | 4.67 | 0.658 | 0.500 |
| O2.Healthy behaviors | 5.00 | 4.71 | 0.463 | 1.000 |
| O3.Motor cognition | 5.00 | 4.38 | 0.740 | 1.000 |
| **Tasks (T)** |  |  |  |  |
| T1.Physical fitness | 5.00 | 4.76 | 0.539 | 0.000 |
| T2.Motor skills | 4.00 | 4.24 | 0.889 | 1.000 |
| T3.Body health | 5.00 | 4.67 | 0.577 | 1.000 |
| T4.Psychological health | 5.00 | 4.71 | 0.561 | 0.500 |
| T5.Rule awareness | 5.00 | 4.52 | 0.750 | 1.000 |
| T6.Safety awareness | 5.00 | 4.81 | 0.680 | 0.000 |
| T7.Teamwork awareness | 4.00 | 4.38 | 0.590 | 1.000 |
| **Indicators (I)** |  |  |  |  |
| I1.Body coordination | 5.00 | 4.62 | 0.590 | 1.000 |
| I2.Quality of velocity | 4.00 | 4.14 | 0.793 | 1.500 |
| I3.Balance | 5.00 | 4.52 | 0.750 | 1.000 |
| I4.Quality of strength | 4.00 | 4.05 | 0.865 | 1.500 |
| I5.Quality of endurance | 4.00 | 3.86 | 0.727 | 1.000 |
| I6.Body movement skills | 5.00 | 4.52 | 0.602 | 1.000 |
| I7.Object control skills | 5.00 | 4.43 | 0.676 | 1.000 |
| I8.Body stability skills | 5.00 | 4.33 | 0.856 | 1.000 |
| I9.Physical activity | 5.00 | 4.67 | 0.483 | 1.000 |
| I10.Motor behavior | 5.00 | 4.38 | 0.740 | 1.000 |
| I11.Emotional mastery | 5.00 | 4.52 | 0.680 | 1.000 |
| I12.self-recognition | 4.00 | 4.33 | 0.730 | 1.000 |
| I13.Dare to challenge | 5.00 | 4.48 | 0.873 | 1.000 |
| I14.Friendly competition | 4.00 | 4.33 | 0.658 | 1.000 |
| I15.Respect for order | 5.00 | 4.67 | 0.658 | 0.500 |
| I16.Self-protection | 5.00 | 4.81 | 0.512 | 0.000 |
| I17.Willingness to cooperate | 4.00 | 4.14 | 0.727 | 1.000 |
| **Contents (C)** |  |  |  |  |
| C1.Hand-eye coordination | 5.00 | 4.76 | 0.436 | 0.500 |
| C2.Hand-foot coordination | 5.00 | 4.57 | 0.507 | 1.000 |
| C3.Reaction velocity | 4.00 | 4.24 | 0.625 | 1.000 |
| C4.Displacement velocity | 4.00 | 4.19 | 0.814 | 1.500 |
| C5.Velocity of body movement | 4.00 | 4.14 | 0.854 | 2.00 |
| C6.Dynamic balance | 5.00 | 4.48 | 0.814 | 1.000 |
| C7.Static balance | 4.00 | 4.24 | 0.889 | 1.000 |
| C8.Upper body Strength | 4.00 | 4.10 | 0.889 | 1.500 |
| C9.Lumbar and abdominal strength | 4.00 | 3.86 | 0.854 | 1.500 |
| C10.Lower body strength | 4.00 | 4.19 | 0.680 | 1.000 |
| C11.Cardiorespiratory endurance | 4.00 | 4.24 | 0.700 | 1.000 |
| C12.Walk | 5.00 | 4.76 | 0.539 | 0.000 |
| C13.Run | 5.00 | 4.86 | 0.359 | 0.000 |
| C14.Skip | 5.00 | 4.86 | 0.359 | 0.000  00  00000 |
| C15.Climb | 5.00 | 4.57 | 0.676 | 1.000 |
| C16.Straddle | 5.00 | 4.38 | 0.740 | 1.000 |
| C17.Slide | 4.00 | 3.90 | 0.889 | 1.000 |
| C18.Racket the ball | 5.00 | 4.43 | 0.746 | 1.000 |
| C19.Hit the ball | 4.00 | 4.10 | 0.889 | 1.500 |
| C20.Passing and receiving the ball | 4.00 | 4.19 | 0.873 | 1.000 |
| C21.Throwing the ball | 4.00 | 4.29 | 0.873 | 1.000 |
| C22.Kick the ball | 4.00 | 4.29 | 0.717 | 1.000 |
| C23.Roll | 4.00 | 4.38 | 0.590 | 1.000 |
| C24.Whirl | 4.00 | 4.10 | 0.944 | 1.000 |
| C25.Hedge | 4.00 | 4.24 | 0.700 | 1.000 |
| C26.Movement instruction | 4.00 | 4.24 | 0.768 | 1.000 |
| C27.Music rhythm | 5.00 | 4.62 | 0.498 | 1.000 |
