# Supplementary material for: The LEG program promotes the development of physical activity and fundamental movement skills in preschool children aged 3–6 years: a Delphi study
Source: Front Public Health. 2025 Mar 25;13:1521878. doi: 10.3389/fpubh.2025.1521878 (PMC11975950; doi:10.3389/fpubh.2025.1521878)
Supplement: Supplementary file 6 [file Table_3.doc]

**Results of Round Three** of the Delphi Study

| **Items** | **Median** | **Mean** | **SD** | **IQD** |
| --- | --- | --- | --- | --- |
| **Objectives (O)** |  |  |  |  |
| O1.Physical capability | 5.00 | 4.60 | 0.821 | 0.750 |
| O2.Healthy behaviors | 5.00 | 4.65 | 0.745 | 0.750 |
| O3.Motor cognition | 5.00 | 4.50 | 0.607 | 1.000 |
| **Tasks (T)** |  |  |  |  |
| T1.Physical fitness | 5.00 | 4.70 | 0.801 | 0.000 |
| T2.Motor skills | 4.50 | 4.30 | 0.865 | 1.000 |
| T3.Body health | 5.00 | 4.60 | 0.940 | 0.750 |
| T4.Psychological health | 5.00 | 4.55 | 0.999 | 0.750 |
| T5.Rule awareness | 5.00 | 4.55 | 0.826 | 1.000 |
| T6.Safety awareness | 5.00 | 4.75 | 0.910 | 0.000 |
| T7.Teamwork awareness | 4.00 | 4.35 | 0.745 | 1.000 |
| **Indicators (I)** |  |  |  |  |
| I1.Body coordination | 5.00 | 4.60 | 0.754 | 1.000 |
| I2.Quality of velocity | 4.00 | 4.15 | 0.875 | 1.000 |
| I3.Balance | 5.00 | 4.60 | 0.754 | 1.000 |
| I4.Quality of strength | 4.00 | 4.10 | 0.912 | 1.750 |
| I5.Quality of endurance | 4.00 | 3.90 | 0.641 | 0.750 |
| I6.Body movement skills | 5.00 | 4.50 | 0.688 | 1.000 |
| I7.Object control skills | 5.00 | 4.40 | 0.821 | 1.000 |
| I8.Body stability skills | 5.00 | 4.45 | 0.759 | 1.000 |
| I9.Physical activity | 5.00 | 4.65 | 0.587 | 1.000 |
| I10.Motor behavior | 4.50 | 4.35 | 0.813 | 1.000 |
| I11.Emotional mastery | 5.00 | 4.50 | 0.688 | 1.000 |
| I12.self-recognition | 4.50 | 4.35 | 0.813 | 1.000 |
| I13.Dare to challenge | 5.00 | 4.50 | 0.889 | 1.000 |
| I14.Friendly competition | 4.00 | 4.40 | 0.598 | 1.000 |
| I15.Respect for order | 5.00 | 4.60 | 0.995 | 0.000 |
| I16.Self-protection | 5.00 | 4.75 | 0.716 | 0.000 |
| I17.Willingness to cooperate | 4.00 | 4.15 | 0.813 | 1.000 |
| **Contents (C)** |  |  |  |  |
| C1.Hand-eye coordination | 5.00 | 4.70 | 0.733 | 0.000 |
| C2.Hand-foot coordination | 4.00 | 4.55 | 0.605 | 1.000 |
| C3.Reaction velocity | 4.00 | 4.30 | 0.733 | 1.000 |
| C4.Displacement velocity | 5.00 | 4.20 | 0.834 | 1.750 |
| C5.Dynamic balance | 4.50 | 4.55 | 0.686 | 1.000 |
| C6.Static balance | 4.00 | 4.30 | 0.865 | 1.000 |
| C7.Upper body Strength | 4.00 | 4.15 | 0.813 | 1.000 |
| C8.Lower body strength | 4.00 | 3.90 | 0.852 | 1.750 |
| C9.Cardiorespiratory endurance | 4.00 | 4.15 | 0.813 | 1.000 |
| C10.Walk | 5.00 | 4.30 | 0.657 | 1.000 |
| C11.Run | 5.00 | 4.75 | 0.716 | 0.000 |
| C12.Skip | 5.00 | 4.75 | 0.716 | 0.000 |
| C13.Climb | 5.00 | 4.75 | 0.716 | 0.000 |
| C14.Straddle | 5.00 | 4.55 | 0.826 | 1.000 |
| C15.Slide | 4.00 | 4.35 | 0.875 | 1.000 |
| C16.Racket the ball | 5.00 | 4.05 | 0.759 | 0.750 |
| C17.Hit the ball | 4.00 | 4.50 | 0.688 | 1.000 |
| C18.Passing and receiving the ball | 4.00 | 4.30 | 0.657 | 1.000 |
| C19.Throwing the ball | 4.00 | 4.30 | 0.801 | 1.000 |
| C20.Kick the ball | 4.00 | 4.35 | 0.745 | 1.000 |
| C21.Roll | 4.00 | 4.30 | 0.733 | 1.000 |
| C22.Whirl | 4.00 | 4.40 | 0.598 | 1.000 |
| C23.Hedge | 4.00 | 4.50 | 0.688 | 1.000 |
| C24.Motor instruction | 4.00 | 4.30 | 0.657 | 1.000 |
| C25.Music rhythm | 4.00 | 4.30 | 0.801 | 1.000 |
